# Pre-Operative Predictors for Post-Operative Pneumonia in Aneurysmal Subarachnoid Hemorrhage After Surgical Clipping and Endovascular Coiling: A Single-Center Retrospective Study

**DOI:** 10.3389/fneur.2022.893516

**Published:** 2022-06-24

**Authors:** Kexin Yuan, Runting Li, Yahui Zhao, Ke Wang, Fa Lin, Junlin Lu, Yu Chen, Li Ma, Heze Han, Debin Yan, Ruinan Li, Jun Yang, Shihao He, Zhipeng Li, Haibin Zhang, Xun Ye, Hao Wang, Hongliang Li, Linlin Zhang, Guangzhi Shi, Jianxin Zhou, Yang Zhao, Yukun Zhang, Youxiang Li, Shuo Wang, Xiaolin Chen, Yuanli Zhao, Qiang Hao

**Affiliations:** ^1^Department of Neurosurgery, Beijing Tiantan Hospital, Capital Medical University, Beijing, China; ^2^Department of Critical Care Medicine, Beijing Tiantan Hospital, Capital Medical University, Beijing, China; ^3^Department of Neurosurgery, Peking University International Hospital, Beijing, China; ^4^Department of Interventional Neuroradiology, Beijing Tiantan Hospital, Capital Medical University, Beijing, China; ^5^China National Clinical Research Center for Neurological Diseases, Beijing, China; ^6^Stroke Center, Beijing Institute for Brain Disorders, Beijing, China; ^7^Beijing Key Laboratory of Translational Medicine for Cerebrovascular Disease, Beijing, China

**Keywords:** aneurysmal subarachnoid hemorrhage, post-operative pneumonia, risk factors, endovascular coiling, surgical clipping

## Abstract

**Objective:**

Postoperative pneumonia (POP) is one of the major complications after aneurysmal subarachnoid hemorrhage (aSAH) associated with postoperative mortality, prolonged hospitalization, and increased medical cost. Early recognition of pneumonia and more aggressive management may improve patient outcomes.

**Methods:**

We retrospectively reviewed all patients with aSAH who were admitted to our institution between January 2015 and December 2020. Baseline clinical characteristics, imaging data, and inflammatory biomarkers were reviewed. The risk factors derived from multivariate logistic regression of surgical clipping (SC) and endovascular coiling (EC) were analyzed. The area under the receiver operating characteristic (ROC) curve (AUC) was used to calculate each independent predictor's prediction ability.

**Results:**

A total of 843 patients were enrolled. Compared with patients in the EC group, the incidence of POP was higher in the SC group [143/414 (34.54%) vs. 114/429 (26.57%), *p* = 0.015]. In the EC group, multivariate analysis revealed that age [*p* = 0.001; odds ratio (OR) = 1.04, 95% CI = 1.02–1.07], posterior circulation aneurysms (*p* = 0.021; OR = 2.07, 95% CI = 1.14–3.83), higher neutrophil (NEUT; *p* < 0.001; OR = 1.13, 95% CI = 1.06–1.21), World Federation of Neurosurgical Societies (WFNS) grade 4 or 5 (*p* < 0.001; OR = 4.84, 95% CI = 2.67–8.79), modified Fisher Scale (mFS) grade 3 or 4 (*p* = 0.022; OR = 2.60, 95% CI = 1.15–5.89), and acute hydrocephalus (*p* = 0.048; OR = 1.74, 95% CI = 1.01–3.00) were independent risk factors for POP. In the SC group, multivariate analysis revealed that age (*p* = 0.015; OR = 1.03, 95% CI = 1.01–1.05), WFNS grade 4 or 5 (*p* = 0.037; OR = 1.76, 95% CI = 1.03–3.00), heart disease (*p* < 0.001; OR = 5.02, 95% CI = 2.03–12.45), higher white blood cell (WBC; *p* < 0.001; OR = 1.13, 95% CI = 1.07–1.20), and mFS grade 3 or 4 (*p* = 0.019; OR = 2.34, 95% CI = 1.15–4.77) were independent risk factors for POP.

**Conclusion:**

Patients treated with SC are more likely to develop POP. Comprehensive preoperative evaluation of patients may help physicians to better predict POP and implement preventive measures to improve outcomes.

## Introduction

Aneurysmal subarachnoid hemorrhage (aSAH) is a severe neurosurgical emergency with a high mortality rate of 22–50% (1–3). Even patients receiving surgical clipping (SC) or endovascular coiling (EC) therapy also left approximately one-third of the patients to suffer from being severely disabled and functionally dependent. Early and reliable prediction of the patients' condition after SAH is important in clinical practice for decision-making about treatment options and providing information for the patients and their families (4, 5).

Severe in-hospital complications may affect the prognosis of patients and increase the medical burden on patients' families and countries. Therefore, early identification of complications associated with poor patient prognosis has considerable clinical importance (6, 7). Postoperative pneumonia (POP) is one of the major complications after aSAH surgery and is associated with postoperative mortality, prolonged hospitalization, and increased medical cost (5, 8).

Three large studies have shown that patients with SC had a higher incidence of hospital complications than those with EC, leading to a higher risk of poor discharge outcomes and even long-term disability (9–12). Our team's research showed that POP may have a long-lasting impact on the prognosis of patients (5). Thus, early identification and active prevention of POP become critical. Some studies have tried to identify risk factors associated with POP; however, the two treatment modalities' preoperative indicators related to risk factors for POP have not been reported (13, 14).

This study retrospectively reviewed the preoperative indicators associated with POP in SC and EC groups. Further, we analyzed the potential causes of pneumonia due to these factors to provide clinical evidence for preventing and treating POP.

## Materials and Methods

### Study Design

We retrospectively reviewed the patient data from consecutive patients with aSAH who were admitted to our institution between January 2015 and December 2020. All patient data were from the Long-Term Prognosis of Emergency Aneurysmal Subarachnoid Hemorrhage (LongTEAM) study. The registry is listed at ClinicalTrials.gov (registration no. NCT 04785976).

This study was approved by the Institutional Review Board of Beijing Tiantan Hospital, Capital Medical University (KY 2021-008-01). All participants or their authorized representatives obtained informed consent for clinical analyses. All the analyses were performed by the Declaration of Helsinki and the local ethics policies. Both procedures were performed by specific senior neurosurgeons, with an annual average of more than 250 procedures per neurosurgeon. All patients were managed according to the American Heart Association/American Stroke Association guidelines (15).

### Inclusion and Exclusion Criteria

All patients had angiographically documented aSAH caused by intracranial aneurysm confirmed by computed tomography (CT) or lumbar puncture. The inclusion criteria were as follows: (1) age ≥18 years; (2) single aneurysm; (3) emergency admission without previous aneurysm rupture; (4) only patients treated by clipping or interventional; (5) complete 90-day follow-up; and (6) no missing data. The exclusion criteria were as follows: (1) admitted over 72 h from rupture to the emergency department; (2) other neurological diseases (tumor, vascular malformation, Parkinson's disease, multiple sclerosis, and primary epilepsy), and functional or neurological deficit of the extremities due to any cause; (3) history of neurosurgery before rupture; and (4) treatments, such as external ventricular drainage, lumbar puncture, angiography, intubation, and mechanical ventilation, at other hospitals before presentation to our hospital.

### Procedures

Baseline clinical characteristics and imaging data were reviewed, such as age, sex, location of the ruptured aneurysm, Graeb score, acute hydrocephalus, and medical and medication history. The severity of aSAH was assessed based on the initial World Federation of Neurosurgical Societies (WFNS) grade and the modified Fisher Scale (mFS) grade. We also collected inflammatory markers, such as white blood cell (WBC), systemic inflammation response index (SIRI), neutrophil (NEUT), monocyte (MONO), monocyte-to-lymphocyte ratio (MLR), platelet-to-white blood cell ratio (PWR), platelet-to-neutrophil ratio (PNR), and neutrophil-to-lymphocyte ratio (NLR).

Postoperative clinical complications, such as rebleeding, delayed cerebral ischemia (DCI), seizures, intracranial infection, stress ulcer bleeding, abnormal hepatic function, urinary tract infection, anemia, hypoproteinemia, POP, and deep vein thrombosis (DVT), during hospitalization were collected. The modified Rankin Scale (mRS) scores were collected at discharge and 90 days after discharge.

### Outcome Assessment

The primary outcome was the occurrence of POP. POP was defined as fever, increased WBC and C-reactive protein (CRP) levels, and chest radiograph showed pulmonary infiltrates within 30 days after surgery, which required antibiotic therapy by a surgeon, according to the modified Centers for Disease Control and Prevention criteria (16).

The secondary outcome was the mRS [a stroke outcome scale with scores ranging from 0 (no symptoms) to 6 (dead)] score at discharge and 90 days after discharge (the neurosurgeon followed up with patients *via* telephone or an outpatient appointment 90 days after discharge). Unfavorable outcomes were defined when the mRS score was ≥3.

### Statistical Analysis

Categorical variables were presented as frequency (percentages), and continuous variables were presented as the means ± standard deviations (SD) or median and interquartile range (IQR). In comparing baseline characteristics and outcomes between groups, the Pearson's chi-square test or Fisher's exact test was used to compare categorical variables as appropriate. After testing for normality, continuous variables were analyzed using the independent Student's *t*-test or Mann-Whitney U rank-sum test (as appropriate). According to the diagnosis of POP, we divided the patients into two groups and performed the univariate regression analysis. Only variables with *p* < 0.05 in univariate analysis were entered in multivariate logistic regression analysis, with adjustments for other characteristics, a forward stepwise model was used to identify the independent predictors of POP between groups. The odds ratio (OR) and 95% confidence intervals (CIs) of variables were calculated. The sensitivities and specificities of predictive factors were calculated from the receiver operating characteristic (ROC) curve analyses. The area under the ROC curve (AUC) was calculated to measure each independent predictor's prediction ability. *p* < 0.05 was considered to be statistically significant. Statistical analyses were performed using the R statistical program (R studio; version 3.3.3), SPSS Statistics version 26.0 (IBM Corp.), and GraphPad PRISM 8.3.0 (GraphPad Software Inc.).

## Results

### Patient Characteristics

**Figure 1:**
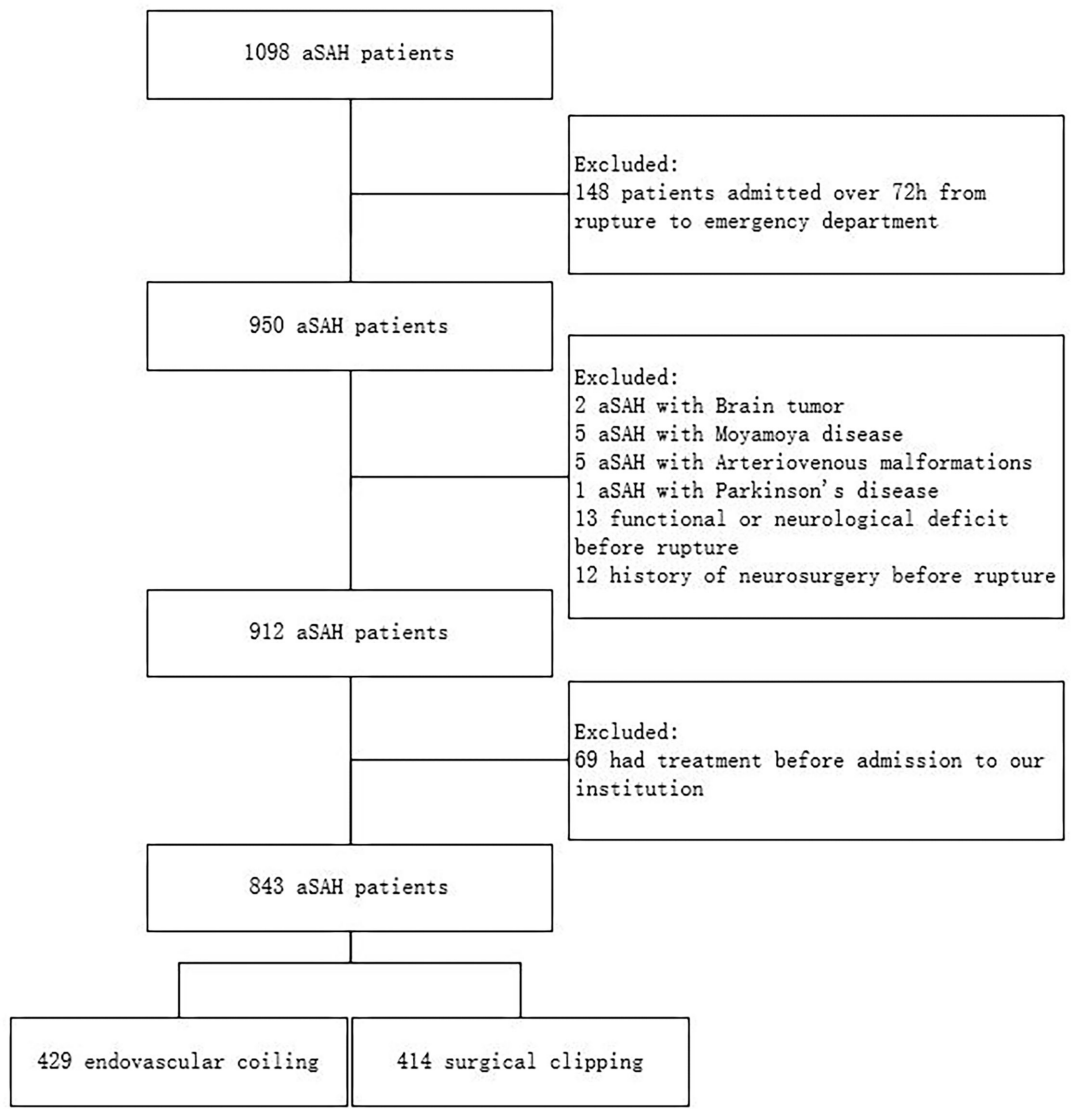
Flowchart of patient selection.

A total of 843 patients in the retrospective cohort who had their hospitalization between January 2015 and December 2020 were enrolled in the present study (Figure 1). Compared with the SC group, patients in the EC group had a higher proportion of female patients [265/429 (61.8%) vs. 226/414 (54.6%), *p* = 0.041] and a higher proportion of posterior circulation aneurysms [77/429 (18.0%) vs. 12/414 (2.9%), *p* < 0.001; Table 1].

**Table 1:** Baseline characteristics and group comparisons between the two treatment modalities.

**Patient characteristics**	**Overall**	**Endovascular coiling**	**Surgical clipping**	***P***
	***N* = 843**	***N* = 429**	***N* = 414**	
Age, y, mean ± SD	54.7 ± 11.1	55.4 ± 12.0	54.0 ± 10.2	0.067
Female, *n* (%)	491 (58.2)	265 (61.8)	226 (54.6)	0.041
WFNS grade 4–5, *n* (%)	182 (21.6)	87 (20.3)	95 (23.0)	0.391
mFS grade 3–4, *n* (%)	661 (78.4)	325 (75.8)	336 (81.2)	0.069
Intraventricular hemorrhage, *n* (%)	525 (62.3)	267 (62.2)	258 (62.3)	>0.99
Acute hydrocephalus, *n* (%)	338 (40.1)	180 (42.0)	158 (38.2)	0.292
Posterior circulation, *n* (%)	89 (10.6)	77 (18.0)	12 (2.9)	<0.001
Current smoking, *n* (%)	244 (29.0)	127 (29.6)	117 (28.3)	0.724
Current alcohol abuse, *n* (%)	187 (22.2)	104 (24.2)	83 (20.1)	0.167
Diabetes mellitus, *n* (%)	82 (9.7)	44 (10.3)	38 (9.2)	0.681
Hypertension, *n* (%)	510 (60.5)	273 (63.6)	237 (57.3)	0.068
Hyperlipidemia, *n* (%)	76 (9.0)	41 (9.6)	35 (8.5)	0.661
Heart disease, *n* (%)	67 (8.0)	40 (9.3)	27 (6.5)	0.169
Current use of anti-platelet aggregation drugs, *n* (%)	43 (5.1)	24 (5.6)	19 (4.6)	0.613

*WFNS, World Federation of Neurological Societies; mFS, modified Fisher Scale*.

### In-Hospital Complications

Compared with the EC group, patients in the SC group had higher incidences of DCI [136/414 (32.9%) vs. 90/429 (21.0%), *p* < 0.001], intracranial infection [83/414 (20.25%) vs. 10/429 (2.33%), *p* < 0.001], anemia [173/414 (41.79%) vs. 85/429 (19.81%), *p* < 0.001], hypoproteinemia [194/414 (46.86%) vs. 94/429 (21.91%), *p* < 0.001], and POP [143/414 (34.54%) vs. 114/429 (26.57%), *p* = 0.015] and a lower incidence of urinary tract infection [4/414 (0.97%) vs. 19/429 (4.43%), *p* = 0.004; Table 2]. The SC group had a higher incidence of unfavorable outcome at discharge [201/414 (48.55%) vs. 150/429 (34.97%), *p* < 0.001] and 90 days after discharge [92/414 (22.22%) vs. 62/429 (14.45%), *p* < 0.001].

**Table 2:** The comparison of in-hospital complications between two treatment modalities.

**Patient characteristics**	**Overall**	**Endovascular coiling**	**Surgical clipping**	***p***
	***N* = 843**	***N* = 429**	***N* = 414**	
Rebleeding, *n* (%)	5 (0.59)	4 (0.93)	1 (0.24)	0.391
Delayed cerebral ischemia, *n* (%)	226 (26.81)	90 (20.98)	136 (32.85)	<0.001
Seizures, *n* (%)	9 (1.07)	5 (1.17)	4 (0.97)	>0.99
Intracranial infection, *n* (%)	93 (11.03)	10 (2.33)	83 (20.05)	<0.001
Stress ulcer bleeding, *n* (%)	192 (22.78)	104 (24.24)	88 (21.26)	0.341
Abnormal hepatic function, *n* (%)	244 (28.94)	116 (27.04)	128 (30.92)	0.244
Urinary tract infection, *n* (%)	23 (2.73)	19 (4.43)	4 (0.97)	0.004
Anemia, *n* (%)	258 (30.60)	85 (19.81)	173 (41.79)	<0.001
Hypoproteinemia, *n* (%)	288 (34.16)	94 (21.91)	194 (46.86)	<0.001
Postoperative pneumonia, *n* (%)	257 (30.49)	114 (26.57)	143 (34.54)	0.015*
Deep vein thrombosis, *n* (%)	73 (8.66)	32 (7.46)	41 (9.90)	0.254
mRS 3–6 at discharge, *n* (%)	351 (41.64)	150 (34.97)	201 (48.55)	<0.001
mRS 3–6 90 days after discharge, *n* (%)	154 (18.27)	62 (14.45)	92 (22.22)	0.005

*P <0.05, significant difference.

### Independent Risk Factors Associated With POP in All Patients

Multivariate analysis showed that age (*p* < 0.001; OR = 1.03, 95% CI = 1.01–1.04), female patients (*p* = 0.012; OR = 1.53, 95% CI = 1.10–2.14), WFNS grade 4–5 (*p* < 0.001; OR = 4.43, 95% CI = 3.09–6.37), mFS grade 3 or 4 (*p* < 0.001; OR = 3.04, 95% CI = 1.82–5.07), and SC (*p* = 0.031; OR = 1.43, 95% CI = 1.03–1.98) were independently associated with POP (Table 3).

**Table 3:** Independent risk factors associated with postoperative pneumonia (POP) in all patients (*N* = 843).

**Variables**	**OR (95%CI)**	***p***
Age	1.03 (1.01–1.04)	<0.001
Female	1.53 (1.10–2.14)	0.012
WFNS grade 4–5	4.43 (3.09–6.37)	<0.001
mFS grade 3–4	3.04 (1.82–5.07)	<0.001
Surgical clipping	1.43 (1.03–1.98)	0.031

*OR, odds ratio; CI, confidence interval; WFNS, World Federation of Neurological Societies; mFS, modified Fisher Scale*.

### Patient Characteristics in the EC Group

Patients who had POP in the EC group were more likely to have intraventricular hemorrhage on admission [94/114 (82.46%) vs. 173/315 (54.92%), *p* < 0.001], a higher incidence of WFNS grade 4 or 5 [57/114 (50.00%) vs. 30/315 (9.52%), *p* < 0.001], a higher incidence of mFS grade 3 or 4 [106/114 (92.98%) vs. 219/315 (69.52%), *p* < 0.001], a higher incidence of acute hydrocephalus [106/114 (62.28%) vs. 109/315 (34.60%), *p* < 0.001], a higher incidence of posterior circulation [33/114 (28.95%) vs. 44/315 (13.97%), *p* < 0.001], a higher incidence of hypertension [88/114 (77.19%) vs. 185/315 (58.73%), *p* < 0.001], a higher incidence of heart disease [19/114 (16.67%) vs. 21/315 (6.67%), *p* = 0.003], and higher level of inflammatory biomarkers, such as WBC [14.94 (11.32–17.96) vs. 11.55 (9.32–14.39), *p* < 0.001], MONO [0.45 (0.30–0.67) vs. 0.36 (0.25–0.54), *p* < 0.001], NEUT [13.10 (10.02–16.19) vs. 10.20 (7.63–12.91), *p* < 0.001], SIRI [6.17 (3.58–10.32) vs. 3.56 (2.25–5.77), *p* < 0.001], MLR [0.47 (0.35–0.67) vs. 0.36 (0.26–0.51), *p* < 0.001], a lower level of PWR [15.98 (13.17–22.74) vs. 19.58 (15.76–23.47), *p* = 0.004], and a lower level of PNR [17.95 (14.61–19.65) vs. 22.44 (17.50–28.40), *p* = 0.002; Table 4).

**Table 4:** Baseline characteristics and inflammatory biomarkers in the endovascular coiling EC group.

**Variable**	**Overall**	**No-POP**	**POP**	***p***
	***N* = 429**	***N* = 315**	***N* = 114**	
Age, y, mean ± SD	55.4 ± 12.0	54.1 ± 11.8	58.8 ± 11.9	<0.001*
Female, *n* (%)	265 (61.8)	201 (63.8)	64 (56.1)	0.183
WFNS grade 4–5, *n* (%)	87 (20.3)	30 (9.5)	57 (50.0)	<0.001*
mFS grade 3–4, *n* (%)	325 (75.8)	219 (69.5)	106 (93.0)	<0.001*
Graeb 5–12, *n* (%)	40 (9.3)	15 (4.76)	25 (21.9)	<0.001*
Acute hydrocephalus, *n* (%)	180 (42.0)	109 (34.6)	71 (62.3)	<0.001*
Posterior circulation, *n* (%)	77 (18.0)	44 (14.0)	33 (29.0)	<0.001*
Current smoking, *n* (%)	127 (29.6)	92 (29.2)	35 (30.7)	0.857
Current alcohol abuse, *n* (%)	104 (24.2)	74 (23.5)	30 (26.3)	0.635
Diabetes mellitus, *n* (%)	44 (10.3)	30 (9.5)	14 (12.3)	0.515
Hypertension, *n* (%)	273 (63.6)	185 (58.7)	88 (77.2)	<0.001*
Hyperlipidemia, *n* (%)	41 (9.6)	30 (9.5)	11 (9.7)	>0.99
Heart disease, *n* (%)	40 (9.3)	21 (6.7)	19 (16.7)	0.003*
Current use of anti-platelet aggregation drugs, *n* (%)	24 (5.6)	16 (5.1)	8 (7.0)	0.594
White blood cell count^a^, median (IQR)	12.4 (9.67–15.53)	11.55 (9.32–14.39)	14.94 (11.32–17.96)	<0.001*
Lymphocyte count^a^, median (IQR)	0.96 (0.70–1.34)	0.98 (0.69–1.33)	0.94 (0.71–1.36)	0.649
Monocyte count^a^, median (IQR)	0.39 (0.27–0.57)	0.36 (0.25–0.54)	0.45 (0.30–0.67)	<0.001*
Neutrophil count^a^, median (IQR)	11.03 (8.17–13.81)	10.20 (7.63–12.91)	13.10 (10.02–16.19)	<0.001*
Platelet count^a^, median (IQR)	228.50 (183.75–269.50)	226.50 (181.00 −262.25)	232.50 (195.25–292.25)	0.028*
SIRI, median (IQR)	4.24 (2.44–7.02)	3.56 (2.25–5.77)	6.17 (3.58–10.32)	<0.001*
MLR, median (IQR)	0.26 (0.20–0.32)	0.36 (0.26–0.51)	0.47 (0.35–0.67)	<0.001*
PWR, median (IQR)	18.36 (14.52–23.25)	19.58 (15.76–23.47)	15.98 (13.17–22.74)	0.004*
PNR, median (IQR)	21.17 (16.17–27.33)	22.44 (17.50–28.40)	17.95 (14.61–19.65)	0.002*
NLR, median (IQR)	11.52 (7.61–17.05)	10.37 (6.12–15.72)	13.60 (9.47–19.65)	0.215

*POP, postoperative pneumonia; mRS, modified Rankin scale; SD, standard deviation; IQR, interquartile range; WFNS, World Federation of Neurological Societies; mFS, modified Fisher Scale; SIRI, systemic inflammation response index; MLR, monocyte-to-lymphocyte ratio; PWR, platelet-to-white blood cell ratio; PNR, platelet-to-neutrophil ratio; NLR, neutrophil-to-lymphocyte ratio*.

^a^*Unit of measurement: 10^9^/L*.

*p <0.05.

### Patient Characteristics in the SC Group

Patients who had POP in the SC group were more likely to have intraventricular hemorrhage on admission [102/143 (71.33%) vs. 156/271 (57.56%), *p* = 0.008], a higher incidence of WFNS grade 4 or 5 [52/143 (36.36%) vs. 43/271 (15.87%), *p* < 0.001], a higher incidence of mFS grade 3 or 4 [131/143 (91.61%) vs. 205/271 (75.65%), *p* < 0.001], a higher incidence of heart disease 19/143 (13.29%) vs. 8/271 (2.95%), *p* < 0.001], a higher incidence of current use of anti-platelet aggregation drugs [12/143 (8.39%) vs. 7/271 (2.58%), *p* = 0.015], and higher level of inflammatory biomarkers, such as WBC [13.59 (11.51–17.37) vs. 11.57 (8.78–14.31), *p* < 0.001], MONO [0.47 (0.32–0.74) vs. 0.40 (0.27–0.56), *p* < 0.001], NEUT [11.60 (9.87–16.00) vs. 10.18 (7.52–12.93), *p* < 0.001], SIRI [5.44 (3.23–10.49) vs. 3.43 (2.34–6.24), *p* < 0.001], MLR [0.50 (0.32–0.73) vs. 0.38 (0.25–0.55), *p* < 0.001], PNR [18.29 (13.92–23.39) vs. 22.21 (17.02–28.98), *p* = 0.005], NLR [12.71 (8.56–20.33) vs. 11.06 (6.32–15.90), *p* = 0.007], and a lower level of PWR [2.00 (1.37–3.15) vs. 19.37 (14.98–23.83), *p* < 0.001; Table 5].

**Table 5:** Baseline characteristics and inflammatory biomarkers in the surgical clipping (SC) group.

**Variable**	**Overall**	**No-POP**	**POP**	**p**
	***N* = 414**	***N* = 271**	***N* = 143**	
Age, y, mean ± SD	54.0 ± 10.2	53.1 ± 10.1	55.7 ± 10.1	0.012*
Female, *n* (%)	226 (54.6)	154 (56.8)	72 (50.4)	0.248
WFNS grade 4–5, *n* (%)	95 (23.0)	43 (15.9)	52 (36.4)	<0.001*
mFS grade 3–4, *n* (%)	336 (81.2)	205 (75.7)	131 (91.6)	<0.001*
Graeb 5–12, *n* (%)	24 (5.8)	9 (3.3)	15 (10.5)	0.003*
Acute hydrocephalus, *n* (%)	158 (38.2)	97 (35.8)	61 (42.7)	0.207
Posterior circulation, *n* (%)	12 (2.9)	8 (3.0)	4 (2.8)	>0.99
Current smoking, *n* (%)	117 (28.3)	69 (25.5)	48 (33.6)	0.104
Current alcohol abuse, *n* (%)	83 (20.1)	51 (18.8)	32 (22.4)	0.465
Diabetes mellitus, *n* (%)	38 (9.2)	20 (7.4)	18 (12.6)	0.117
Hypertension, *n* (%)	237 (57.3)	147 (54.2)	90 (62.9)	0.111
Hyperlipidemia, *n* (%)	35 (8.5)	27 (10.0)	8 (5.6)	0.182
Heart disease, *n* (%)	27 (6.5)	8 (3.0)	19 (13.3)	<0.001*
Current use of anti-platelet aggregation drugs, *n* (%)	19 (4.6)	7 (2.6)	12 (8.4)	0.014*
White blood cell count^a^, median (IQR)	12.32 (10.02–15.60)	11.57 (8.78–14.31)	13.59 (11.51–17.37)	<0.001*
Lymphocyte count^a^, median (IQR)	0.97 (0.70–1.40)	0.98 (0.69–1.46)	0.96 (0.70–1.40)	0.252
Monocyte count^a^, median (IQR)	0.43 (0.29–0.60)	0.40 (0.27–0.56)	0.47 (0.32–0.74)	<0.001*
Neutrophil count^a^, median (IQR)	10.83 (8.60–13.90)	10.18 (7.52–12.93)	11.60 (9.87–16.00)	<0.001*
Platelet count^a^, median (IQR)	225.00 (194.50–268.50)	224.50 (190.25–267.50)	228.00 (198.50–269.50)	0.413
SIRI, median (IQR)	4.10 (2.61–8.09)	3.43 (2.34–6.24)	5.44 (3.23–10.49)	<0.001*
MLR, median (IQR)	0.40 (0.26–0.61)	0.38 (0.25–0.55)	0.50 (0.32–0.73)	<0.001*
PWR, median (IQR)	18.16 (14.10–23.13)	19.37 (14.98–23.83)	2.00 (1.37–3.15)	<0.001*
PNR, median (IQR)	21.18 (15.88–26.94)	22.21 (17.02–28.98)	18.29 (13.92–23.39)	0.005*
NLR, median (IQR)	11.67 (7.35–17.78)	11.06 (6.32–15.90)	12.71 (8.56–20.33)	0.007*

*POP, postoperative pneumonia; mRS, modified Rankin scale; SD, standard deviation; IQR, interquartile range; WFNS, World Federation of Neurological Societies; mFS, modified Fisher Scale; SIRI, systemic inflammation response index; MLR, monocyte-to-lymphocyte ratio; PWR, platelet-to-white blood cell ratio; PNR, platelet-to-neutrophil ratio; NLR, neutrophil-to-lymphocyte ratio*.

^a^*Unit of measurement: 10^9^/L*.

*p <0.05.

### Independent Risk Factors Associated With POP in the EC Group

Multivariate analysis showed that age (*p* = 0.001; OR = 1.04, 95% CI = 1.02–1.07), posterior circulation aneurysms (*p* = 0.021; OR = 2.07, 95% CI = 1.14–3.83), higher NEUT (*p* < 0.001; OR = 1.13, 95% CI = 1.06–1.21), WFNS grade 4 or 5 (*p* < 0.001; OR = 4.84, 95% CI = 2.67–8.80), mFS grade 3 or 4 (*p* = 0.022; OR = 2.60, 95% CI = 1.15–5.89), and acute hydrocephalus (*p* = 0.048; OR = 1.74, 95% CI = 1.01–3.00) were independently associated with POP in the EC group (Table 6).

**Table 6:** Independent risk factors associated with postoperative pneumonia (POP) in the endovascular coiling (EC) and surgical clipping (SC) groups.

**Variables**	**OR (95%CI)**	**p**
**Independent risk factors associated with POP in the EC groups**		
Age	1.04 (1.02–1.07)	0.001
Posterior circulation	2.07 (1.12–3.83)	0.021
NEUT	1.13 (1.06–1.21)	<0.001
WFNS grade 4–5	4.84 (2.67–8.79)	<0.001
mFS grade 3–4	2.60 (1.15–5.89)	0.022
Acute hydrocephalus	1.74 (1.01–3.00)	0.048
**Independent risk factors associated with POP in the SC groups**		
Age	1.03 (1.01–1.05)	0.015
Heart disease	5.02 (2.03–12.45)	<0.001
WFNS grade 4–5	1.76 (1.03–3.00)	0.037
WBC	1.13 (1.07–1.20)	<0.001
mFS grade 3–4	2.34 (1.15–4.77)	0.019

*OR, odds ratio; CI, confidence interval; NEUT, neutrophil; WBC, white blood cell; WFNS, World Federation of Neurological Societies; mFS, modified Fisher Scale*.

### Independent Risk Factors Associated With the Assurance of POP in the SC Group

Multivariate analysis showed that age (*p* = 0.015; OR = 1.03, 95% CI = 1.01–1.05), WFNS grade 4 or 5 (*p* = 0.037; OR 1.76, 95% CI 1.03–3.00), heart disease (*p* < 0.001; OR = 5.02, 95% CI = 2.03–12.45), higher WBC (*p* < 0.001; OR = 1.13, 95% CI = 1.07–1.20), and mFS grade 3 or 4 (*p* = 0.019; OR = 2.34, 95% CI = 1.15–4.77) were independently associated with POP in the SC group (Table 5).

### ROC Curve Analysis

**Figure 2:**
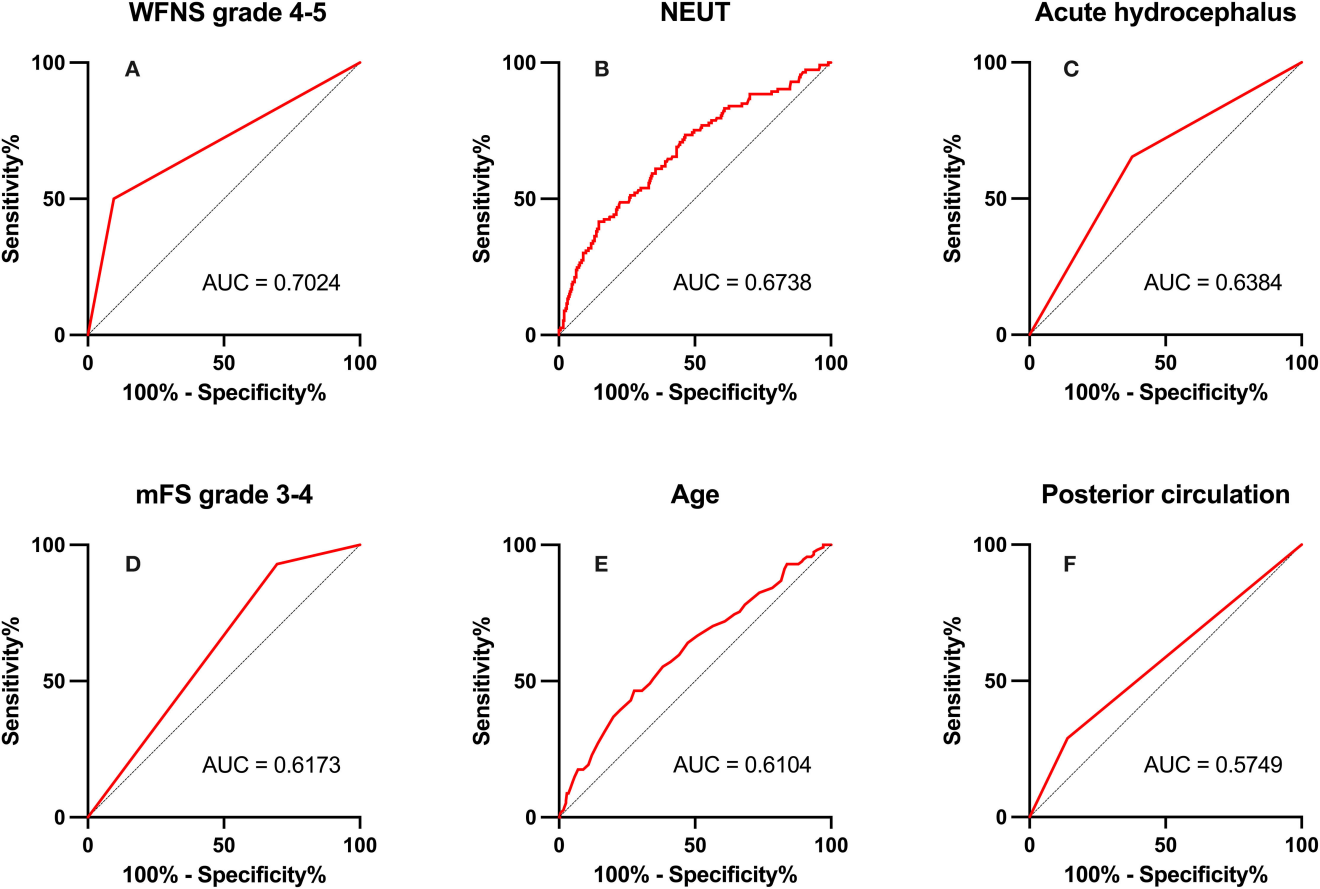
Area under the receiver operating characteristic (AUC) values for preoperative factors that predicted postoperative pneumonia in the endovascular coiling (EC) group. **(A)** World Federation of Neurosurgical Societies (WFNS) grade 4–5 (AUC = 0.702, 95% CI = 2.67–8.80; *p* < 0.001). **(B)** Neutrophil (NEUT; AUC = 0.674, 95% CI = 1.06–1.22; *p* < 0.001). **(C)** Acute hydrocephalus (AUC = 0.638, 95% CI = 1.01–3.00; *p* = 0.048). **(D)** modified Fisher Scale (mFS) grade 3–4 (AUC = 0.617, 95% CI = 1.15–5.89; *p* = 0.022). **(E)** Age (AUC = 0.610, 95% CI = 1.02–1.07; *p* = 0.001). **(F)** Posterior circulation (AUC = 0.575, 95% CI = 1.12–3.83; *p* = 0.021).

**Figure 3:**
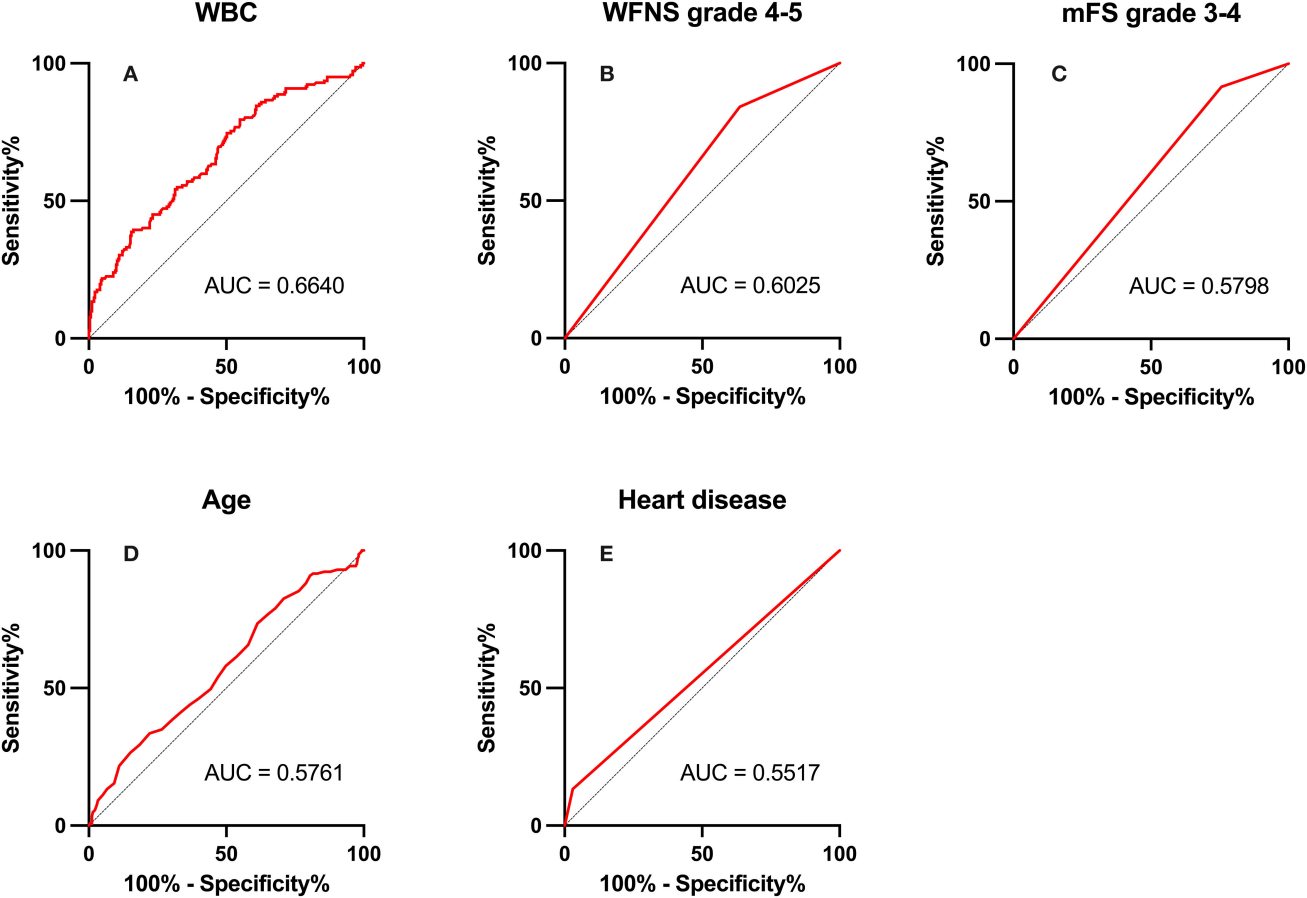
Area under the receiver operating characteristic (AUC) values for preoperative factors that predicted postoperative pneumonia in the surgical clipping (SC) group. **(A)** White blood count (WBC; AUC = 0.664, 95% CI = 1.07–1.20; *p* < 0.001). **(B)** World Federation of Neurosurgical Societies (WFNS) grade 4–5 (AUC = 0.603, 95% CI = 1.03–3.00; *p* = 0.037). **(C)** Modified Fisher Scale (mFS) grade 3–4 (AUC = 0.580, 95% CI = 1.15–4.77; *p* = 0.019). **(D)** Age (AUC = 0.576, 95% CI = 1.01–1.05; *p* = 0.015). **(E)** Heart disease (AUC = 0.552, 95% CI = 2.03–12.45; *p* < 0.001).

In ROC analysis, when the AUC of a variable was >0.7, the predictor variable was defined as a good predictor. The AUC values for each independent risk factor that predicts POP in the SC group and EC group are shown in Figures 2, 3, respectively.

Among preoperative indicators, WFNS grade 4 or 5 showed good predictive ability for POP in the EC group (AUC = 0.702, 2.67–8.80; *p* < 0.001).

## Discussion

Postoperative pneumonia is one of the major complications after aSAH surgery associated with postoperative mortality, prolonged hospitalization, and increased medical cost (6, 17–19). Our previous study revealed that among in-hospital complications, the occurrence of POP showed good predictive efficacy of 90-day unfavorable outcomes in the SC group and EC group (AUC > 0.7) (5). Therefore, trying to identify the occurrence of POP at an early stage is of great significance to improve patient outcomes.

In previous studies, SC was an independent risk factor for the occurrence of POP after aSAH (13). Therefore, we should pay more attention to the baseline characteristics and laboratory tests before the occurrence of POP after SC and EC to predict the occurrence of POP in advance and take preventive measures in time. Unlike previous studies, this study paid more attention to distinguish between groups with different treatment modalities. Our study showed that age, WFNS grade 4 or 5, and mFS grade 3 or 4 were common independent risk factors associated with POP in both groups.

Aspiration of oropharyngeal fluid containing pathogenic microorganisms is the most common cause of POP (20). In addition, the more bacteria inhaled, the greater the likelihood of aspiration pneumonia (21, 22). Most aSAH operations are emergency operations with inadequate preoperative preparation, and patients often have symptoms of vomiting. Preoperative respiratory exercise and oral care are rarely performed, resulting in more oral bacteria and an increased risk of POP. With the increase in age, the immune function of the human body gradually decreases, the tracheal ciliary protective movement and cough reflex become worse, and the bacterial colonization of the oral and upper respiratory tract is significantly increased, leading to a significant increase in the risk of aspiration. At the same time, the increase in age will also lead to organ function failure, resulting in slower drug metabolism after general anesthesia, prolonged neuromuscular block time after anesthesia, and increased the probability of aspiration, ultimately leading to an increased incidence of POP (23, 24). Patients with a higher initial WFNS grade are more likely to stay in the hospital longer, increasing the probability of aspiration, which in turn increases the potential risk of POP. Previous studies have shown that poor postoperative awareness may be a risk factor for poor prognosis, which may also be partly related to the increased incidence of POP (14). Meanwhile, we analyzed the correlation between WFNS grade and inflammatory indicators and found that WBC and NEUTs were significantly higher in WFNS grade 4–5 patients than in WFNS grade 1–3 patients, that is to say, patients with high WFNS grade may have a more severe inflammatory response.

**Figure 4:**
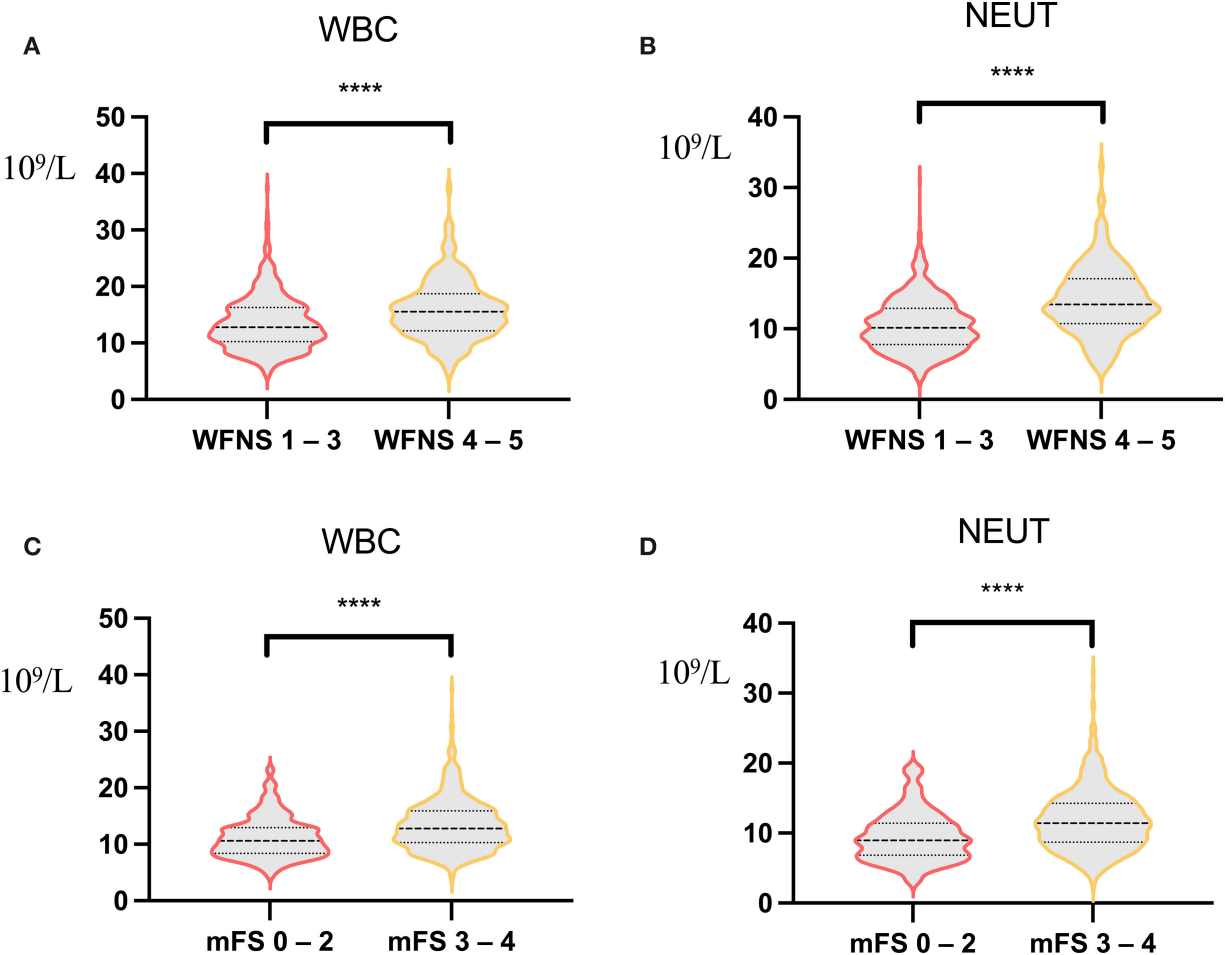
Correlation analysis of World Federation of Neurosurgical Societies (WFNS) score, modified Fisher Scale (mFS) score, and inflammatory indicators. **(A)** Correlation analysis of white blood count (WBC) between WFNS grade 1–3 and WFNS grade 4–5 [12.14 (3.88) vs. 15.88 (5.19), *p* < 0.001]. **(B)** Correlation analysis of neutrophil between WFNS grade 1–3 and WFNS grade 4–5 [10.59 (3.78) vs. 14.03 (4.82), *p* < 0.001]. **(C)** Correlation analysis of WBC between mFS grade 0–2 and mFS grade 3–4 [11.18 (3.59) vs. 13.44 (4.57), *p* < 0.001]. **(D)** Correlation analysis of neutrophil between mFS grade 0–2 and mFS grade 3–4 [9.50 (3.52) vs. 11.85 (4.31), *p* < 0.001). ****p < 0.001.

Studies in mouse models have shown that NEUTs may be important mediators of early cortical hypoperfusion and oxidative stress after aSAH (25). Depletion of NEUTs 3 days after SAH mitigates tissue inflammation and reverses cerebral vasoconstriction in the middle cerebral artery (26); thus, NEUTs are closely associated with oxidative stress and cerebral vasoconstriction. Increased NEUTs or WBCs in the blood before surgery may set the stage for a more intense early systemic inflammatory response in the early stages of brain injury, increasing susceptibility to systemic infections, such as POP. Meanwhile, our data showed that patients with high WFNS grade or high mFS grade had a severe inflammatory response (Figure 4).

Hydrocephalus caused by aSAH can also manifest as high cranial pressure, which can lead to Cushing's reaction (CR). CR compensates for hypothalamic-mediated acute hyperemic response to intracranial pressure (ICP) increment. In patients with aSAH, CR mainly presents as sympathetic peaks with increased serum epinephrine and/or norepinephrine levels. High levels of these two hormones mediate early lymphocyte activation defects leading to POP (27). The severity of CR at SAH depends on the height of ICP and depends on the amount of blood that ruptured into the subarachnoid space (20, 28). Patients with high mFS grade may develop a persistent and excessive systemic inflammatory response syndrome that leads to immunosuppression and is more prone to POP.

Since posterior circulation aneurysms originate anteriorly in the brain stem, rupture and bleeding of posterior circulation aneurysms may affect the brain stem. Both the International Subarachnoid Aneurysm Trial (ISAT) (9) and International study of Unrupted intracranial Aneurysms (ISUIA) (29) studies confirmed that the complication rate of SC for posterior circulation aneurysms is much higher than that of EC treatment. The posterior circulation aneurysm in our center is also mainly treated by EC. At the same time, posterior circulation aneurysms are more likely than other aneurysms to cause an increase in ICP and hydrocephalus. In this case, the EC treatment itself does not improve the condition of hydrocephalus. All of these aggravated CR and increased the risk of POP. Rupture of posterior circulation aneurysm can also increase the incidence of cerebral vasospasm, which affects the cerebral nerves in the posterior approach group, thereby increasing the risk of vomiting and aspiration, leading to an increased incidence of POP (30). Patients with posterior circulation aneurysms with other risk factors of POP, such as a high WFNS grade and a high mFS grade, can be considered an early routine ICP reduction, such as lumbar cistern drainage, in addition to EC therapy.

For patients with pre-existing heart disease, cardiac dysfunction will lead to decreased lung function, significantly increase the probability of cardiogenic pulmonary edema, seriously impair lung ventilation and ventilation function, and reduce the ability of the lung to fight infection (31). They are more likely to develop POP after a traumatic craniotomy.

Therefore, we should pay more attention to preoperative indicators, especially preoperative laboratory indicators, such as WBC and NEUT, which may be of great significance for the identification of early POP. More prospective trials should be conducted in the future to confirm our results.

For patients who underwent elective coronary artery bypass-grafting surgery, a previous study has proved that taking some preventive, tailored interventions to improve inspiratory muscle function can reduce the incidence of POP by 50%. This raises the possibility of preoperatively identifying patients at high risk for POP and taking preventive measures to reduce the incidence of POP after surgery (32). However, aSAH is mostly emergency surgery, which makes our time even more stressful. Advances in surgical techniques have improved patient outcomes, meanwhile, another challenge is the periprocedural management of patients for anesthesiologists and intensivists (33). At present, there is no recognized effective measure to prevent the occurrence of POP. This is also the goal of our further study, starting with preoperative inflammatory parameters and serum epinephrine and/or norepinephrine levels to reduce the secondary attack of surgery on the lungs. We also plan to collect prospective data and build a scoring model for pneumonia in further studies to make it easier for all newly admitted patients to identify the potential risk of pneumonia as early as possible.

Once associated preoperative risk factors are identified, patients at high risk should take targeted preventive measures, such as quitting smoking as soon as physical examination reveals an unruptured aneurysm, initiating doctor-directed prophylaxis with inhale steroids and bronchodilators at the onset of symptoms, and pay attention to respiratory physical therapy and circulatory management. Strategies to inhibit catecholamine in the hyperacute phase may help to prevent vasospasm and improve patient outcomes (34).

### Limitations

The study has several limitations. First, our data were collected retrospectively. Second, differences in preoperative baseline information between the two groups may lead to different risk factors for POP. Third, our study was a single-center study, lacking multicenter validation.

## Conclusion

In summary, this large, single-center retrospective cohort study demonstrated that patients with aSAH treated with SC are more likely to develop POP. Patients with high preoperative inflammatory factors, high WFNS grade, and high mFS grade should be more alert to the occurrence of POP. Comprehensive preoperative evaluation of patients may help physicians to better predict POP and implement preventive measures to improve their outcomes.

## Data Availability Statement

The raw data supporting the conclusions of this article will be made available by the authors, without undue reservation.

## Ethics Statement

The studies involving human participants were reviewed and approved by Institutional Review Board of Beijing Tiantan Hospital. The patients/participants provided their written informed consent to participate in this study.

## Author Contributions

QH and YuaZ: conception, design, and reviewed submitted version of manuscript. KY, RunL, FL, YC, JL, HH, DY, RuiL, JY, ZL, HZ, HL, LZ, YanZ, and YukZ: acquisition of data. KY and RunL: drafting the article. KY, RunL, FL, YC, JL, and QH: statistical analysis. YL, SW, GS, and JZ: administrative, technical, and material support. QH, XC, and YuaZ: study supervision. All authors: analysis, interpretation of data, contributed to the article, and approved the submitted version.

## Funding

This study was supported by the National Key Research and Development Program of China (Grant Nos. 2021YFC2501101 and 2020YFC2004701), the National Natural Science Foundation of China (Grant Nos. 82071296, 81671129, and 81471210), the Beijing Municipal Administration of Hospitals Incubating Program, Beijing, China (Grant No. pX2020023), and the Natural Science Foundation of Beijing, China (Grant No. 7204253).

## Conflict of Interest

The authors declare that the research was conducted in the absence of any commercial or financial relationships that could be construed as a potential conflict of interest.

## Publisher's Note

All claims expressed in this article are solely those of the authors and do not necessarily represent those of their affiliated organizations, or those of the publisher, the editors and the reviewers. Any product that may be evaluated in this article, or claim that may be made by its manufacturer, is not guaranteed or endorsed by the publisher.
